# Multi-Stakeholder Perspectives on Barriers to Mental Health Support for Informal Caregivers

**DOI:** 10.3390/ijerph23030325

**Published:** 2026-03-06

**Authors:** Maria Lizette Rangel, Donaji Stelzig, Cassandra Martinez Enriquez, Hoda Badr

**Affiliations:** 1 Department of Medicine, Baylor College of Medicine, Houston, TX 77030, USA; maria.rangel@bcm.edu (M.L.R.); mcassandra777@gmail.com (C.M.E.); 2 Trust CHW Training Center, Houston, TX 77054, USA; ceo@trustchw.org; 3 Department of Medical Oncology, Sidney Kimmel Comprehensive Cancer Center, Thomas Jefferson University, 834 Chestnut Ave Suite 314, Philadelphia, PA 19107, USA

**Keywords:** informal caregivers, mental health support, health services access, community health workers, qualitative research, The Socioecological Model

## Abstract

**Highlights:**

**Public health relevance—How does this work relate to a public health issue?**

- Informal caregiver psychological distress is a widespread public health concern driven in part by inequitable access to mental health services.
- Caregiver experiences reveal how multilevel structural and policy conditions constrain access to mental health support and contribute to inequities.

**Public health significance—Why is this work of significance to public health?**

- The study demonstrates how barriers and limited supports for caregiver mental health operate and reinforce one another across socioecological contexts.
- Findings identify system- and policy-level conditions that shape caregivers’ ability to recognize need, navigate services, and sustain engagement with care.

**Public health implications—What are the key implications or messages for practitioners, policy makers and/or researchers in public health?**

- Practitioners should implement culturally and linguistically responsive, caregiver-focused mental health screening and navigation, including community health workers.
- Policies and research should support stable, caregiver-inclusive mental health services that address structural barriers and strengthen system-level supports.

**Abstract:**

Background: Informal caregivers experience elevated psychological distress but face substantial challenges in accessing mental health support. Prior research has focused primarily on individual or interpersonal determinants, with less attention to how organizational, community, and policy contexts shape access and contribute to inequities in service availability. Methods: Fifty-one stakeholders, including 17 informal caregivers, 23 community health workers (CHWs), and 11 mental health professionals, completed a demographic survey and 60–90-min semi-structured interviews in English or Spanish. Data were analyzed using thematic analysis, combining deductive coding guided by the Socioecological Model with inductive identification of emergent themes. Results: Participants identified both barriers and supports influencing access to caregiver mental health support across socioecological levels. Individual-level barriers included limited mental health literacy, stigma, competing responsibilities, and language or technology challenges. Interpersonal barriers reflected family minimization of distress and limited encouragement for help-seeking. Organizational barriers involved program instability, restrictive eligibility criteria, long wait times, limited cultural responsiveness, and workplace constraints, while community-level influences included stigma and scarce affordable services. Policy-level barriers reflected immigration-related exclusions and the absence of caregiver-specific mental health coverage. Identified supports, such as CHW navigation and culturally responsive services, were often constrained by structural and organizational limitations. Conclusions: Caregivers face intersecting, multilevel barriers that constrain access to mental health support, while available supports are frequently insufficient to overcome structural constraints. Findings highlight the need for coordinated public health and systems-level strategies that address organizational and policy conditions shaping equitable access to caregiver mental health care.

## 1. Introduction

Caregiving is a cornerstone of the U.S. healthcare system, with millions of individuals providing unpaid support to family members, friends, or others with illness, disability, or age-related needs. Informal caregivers are unpaid individuals who assist with daily activities and health management, enabling care recipients to remain in their homes and communities [1]. In 2024, more than 105 million Americans, representing over 40% of the population, reported serving as informal caregivers [2], and this number is expected to grow as demand for long-term care increases. While caregiving supports care delivery and reduces institutional use, it is often associated with financial strain, poorer physical health, and elevated psychological distress. Despite their essential role, caregivers’ mental health needs remain insufficiently addressed and represent a significant public health concern.

Extensive research has documented elevated stress, anxiety, depression, and burnout among caregivers, along with persistent barriers to accessing mental health support. These risks are heightened by limited formal support and barriers such as stigma, time constraints, cost, and lack of awareness of available services [1,3,4]. However, much of the literature relies on convenience samples that overrepresent White, middle-class caregivers, limiting understanding of how racially, ethnically, and socioeconomically diverse caregivers experience barriers to mental health support [5,6,7,8]. Prior research shows that Black caregivers often experience heavier caregiving demands, while Hispanic and Asian American caregivers report greater difficulty navigating healthcare and social services [8]. These disparities highlight the need to examine caregiving and mental health access within broader cultural, linguistic, and structural contexts.

Unmet mental health needs among caregivers have serious consequences for caregivers and care recipients. Chronic psychological distress is linked to higher morbidity, poorer physical functioning, and reduced quality of life. Care recipients supported by distressed caregivers also experience worse treatment adherence, symptom management, and health outcomes [9,10]. Although the consequences of unmet caregiver mental health needs are well documented, mental health services rarely treat caregivers as a distinct population. This reflects broader gaps in health service design that fail to address caregivers’ ongoing mental health needs. Access is further constrained by fragmented systems, long wait times, high out-of-pocket costs, and unequal distribution of services [11,12,13]. These barriers are especially pronounced for caregivers who are older, low-income, or living in under-resourced communities [14,15].

Importantly, prior research has largely framed barriers to caregiver mental health support at the individual or interpersonal level. This work emphasizes coping, family dynamics, and stigma, with less attention to organizational, community, and policy contexts that shape access to care [16,17,18]. This framing implicitly places responsibility for help-seeking on caregivers and obscures the structural conditions that affect need recognition, service navigation, and receipt of care. As a result, the field has limited ability to explain how barriers are produced and sustained across systems. Without a multilevel perspective, interventions risk targeting individual behavior while leaving organizational and policy barriers unaddressed.

To address this gap, this study applies the Socioecological Model to examine barriers to caregiver mental health support across individual, interpersonal, organizational, community, and policy levels [19]. Although widely used in caregiving stress and coping research [20], the model has been underutilized in studies of caregiver mental health care access. The Socioecological Model conceptualizes access as shaped by conditions beyond the individual and provides a framework for identifying breakdowns along the pathway from need recognition to service engagement.

Within this pathway, caregivers’ experiences of access are shaped not only by formal healthcare systems but also by community-based service roles that support navigation and engagement with care. Community health workers (CHWs) are embedded within the communities they serve and provide outreach, trust-building, care navigation, and advocacy, particularly for caregivers facing linguistic, cultural, financial, or structural barriers. Positioned at the intersection of individual experiences and institutional systems, CHWs offer insight into how barriers to mental health support emerge during service navigation and engagement, complementing caregiver and provider perspectives.

This study examines access to mental health support among informal caregivers through interviews with caregivers, community health workers, and mental health professionals. It is designed to explore how factors across multiple socioecological contexts shape caregivers’ ability to recognize mental health needs, navigate services, and engage with care. By incorporating perspectives from multiple roles involved in caregiving and service delivery, the study aims to inform more equitable and responsive approaches to caregiver mental health support and to contribute evidence relevant to public health practice, community-based interventions, and policy development.

## 2. Materials and Methods

### 2.1. Study Design and Context

This qualitative study used semi-structured interviews to examine barriers to mental health support among informal caregivers. Guided by the Socioecological Model, the analysis considered barriers across individual, interpersonal, organizational, community, and policy contexts. The study was conducted in Harris County, Texas, a large and diverse urban area with substantial economic and health care inequities. Approximately 16 percent of residents live below the federal poverty line, and 22 percent lack health insurance [21]. These conditions provide important context for understanding how local service systems and policy environments shape access to mental health support.

Participants included informal caregivers, CHWs, and mental health professionals, reflecting different roles within caregiving and mental health systems. Caregivers described experiences of distress and efforts to seek help. CHWs described challenges related to services and eligibility. Mental health professionals described organizational and policy factors affecting service delivery. Findings are presented together to illustrate how barriers to caregiver mental health support arise across roles within the care system, rather than to compare groups or assign equal weight to their perspectives.

### 2.2. Ethical Considerations

This study was reviewed and approved by the Institutional Review Board of Baylor College of Medicine (Protocol H-53881). Given the minimal-risk nature of the research, the IRB approved a waiver of written informed consent. Participants received an information sheet describing the study purpose, procedures, potential risks, and confidentiality protections, and provided verbal informed consent prior to participation. This consent process was intended to reduce participant burden and protect confidentiality, particularly for individuals with concerns related to immigration status, employment, or mental health stigma. Participation was voluntary, and participants were informed that they could decline to answer any questions or withdraw from the study at any time without penalty. All data were de-identified prior to analysis.

### 2.3. Participants and Recruitment

Participants included informal caregivers, CHWs, and mental health professionals. Caregivers were adults providing unpaid care to an individual with a serious or chronic condition. CHWs and mental health professionals were eligible if their roles involved supporting caregivers or families managing illness. All participants were fluent in English or Spanish.

Purposive and snowball sampling were used to recruit participants with diverse experiences across caregiving roles and service contexts, including caregivers less connected to formal health care systems. Recruitment was conducted in partnership with local community-based organizations, including a CHW training nonprofit, and through the Community Outreach and Engagement Office at the Dan L Duncan Comprehensive Cancer Center. Interested individuals completed an IRB-approved verbal consent process, a brief sociodemographic survey, and a semi-structured interview.

### 2.4. Data Collection

Semi-structured interviews were conducted between September and December 2023 in English or Spanish, based on participant preference, either via videoconference or in person at a community health center. Prior to the interview, participants completed a brief sociodemographic survey. Interviews were conducted one-on-one, lasted approximately 60–90 min, and were audio-recorded and professionally transcribed verbatim in the language spoken. Participants received a USD 75 gift card for participation. Interviewers had formal training in qualitative research methods and experience conducting community-based research with diverse caregiver populations.

The interview guide was informed by the Socioecological Model and designed to elicit experiences related to access to mental health support across multiple levels. At the individual level, questions explored caregiving responsibilities, emotional well-being, and perceived need for mental health support (e.g., “What are the barriers to seeking mental health services or support?”). Interpersonal questions addressed family dynamics, social support, and stigma (e.g., “Are there cultural or interpersonal dynamics that influence how you or caregivers seek help for mental health?”). Organizational questions focused on experiences with health care providers and service access (e.g., “What barriers have you or caregivers encountered in accessing mental health resources or services?”). Community-level questions addressed resource availability and social norms (e.g., “Are there programs or services in your community that support caregiver mental health?”). Policy-level questions examined broader systemic influences, including insurance coverage, eligibility criteria, and immigration-related concerns (e.g., “Have any rules or policies made it harder for you or caregivers to get help?”). Open-ended questions and probes were used to encourage elaboration and allow participants to frame experiences in their own terms. Field notes were recorded during and immediately following interviews to capture contextual observations and preliminary analytic insights.

Given the sensitive nature of caregiving strain and mental health discussions, interviewers attended to participants’ emotional cues and balanced empathetic engagement with adherence to the interview guide. Reflexivity was supported through regular team debriefings, during which interview experiences, emerging interpretations, and potential assumptions were discussed and used to refine analytic focus.

Recruitment and analysis occurred concurrently and continued until thematic saturation was reached, defined as the point at which additional interviews did not yield new codes or substantively new themes relevant to barriers to caregiver mental health support. Given the multi-stakeholder design, saturation was assessed within each stakeholder group rather than across the combined sample. Preliminary coding and analytic memo-writing were conducted throughout data collection to assess whether new interviews contributed additional conceptual content.

### 2.5. Data Analysis

Data were analyzed using thematic content analysis, supported by ATLAS.ti (Version 24). Analysis was guided by a codebook structured around the five levels of the Socioecological Model [19]. Initial codes were developed deductively based on the conceptual framework and refined through team review of early transcripts. Inductive codes were added to capture themes that extended beyond the initial framework and to ensure analyses reflected participants’ accounts.

Stakeholder perspectives were expected to show both convergence and divergence. Convergence was interpreted as evidence of shared or structurally embedded barriers, while divergence reflected differences in role, access to information, and institutional position. The analytic goal was not to resolve discrepancies but to examine how barriers were experienced across roles. Comparative matrices were used to examine patterns within and across stakeholder groups and socioecological levels. Stakeholder groups were treated as analytically complementary rather than equally weighted sources of evidence. Although findings are presented in aggregate to highlight system-level barriers, caregivers primarily informed individual and interpersonal themes, CHWs informed organizational and community-level constraints, and mental health professionals informed policy and service delivery contexts.

Two members of the research team (MLR, CM) independently coded all transcripts. Coders completed a calibration process prior to full coding to ensure shared understanding of code definitions. Coding discrepancies were resolved through discussion, with adjudication by the Principal Investigator (HB) when consensus could not be reached. Memo-writing and audit trails were maintained throughout analysis to document analytic decisions, codebook revisions, and saturation judgments. Saturation decisions were discussed during regular team meetings. Descriptive statistics from the sociodemographic survey were generated to characterize the sample and provide contextual grounding for the qualitative findings.

### 2.6. Reflexivity and Researcher Positionality

The research team considered how their training, institutional affiliation, and prior relationships with community organizations could influence data collection and interpretation. Team members were trained in public health, behavioral science, and health services research and were affiliated with a comprehensive cancer center. Several team members had prior collaborative relationships with community organizations through outreach and engagement activities; however, no interviewers held supervisory or evaluative authority over participants. To address potential bias and power dynamics, the team clarified research roles, emphasized voluntary participation, used culturally responsive language, maintained reflexive field notes, and held regular debriefings. These practices informed analytic decisions, including revisiting transcripts and refining inductive codes to ensure interpretations remained grounded in participants’ accounts.

## 3. Results

A total of 51 stakeholders participated in the study, including 17 informal caregivers, 23 community health workers (CHWs), and 11 mental health professionals. As shown in Table 1, the sample was predominantly female and racially and ethnically diverse, with nearly three-quarters identifying as Hispanic and 18% as Black or African American. Participants represented a broad age range and varied socioeconomic backgrounds, with approximately one-third reporting household incomes below USD 25,000.

Caregivers reported providing substantial unpaid care, averaging more than 30 h per week, often over extended periods. More than half had been caregiving for one to three years, and most were caring for a family member with cancer, frequently alongside multiple or complex health conditions. Several caregivers described providing care to more than one individual.

CHWs represented a range of community-based roles supporting families navigating health and social service systems. More than one-third also identified as caregivers themselves, reflecting overlapping professional and caregiving experiences. Mental health professionals worked across diverse practice settings and reported varying levels of training specific to caregiver mental health.

### 3.1. Multilevel Barriers and Supports for Caregiver Mental Health

Thematic analysis showed that access to mental health support for caregivers was shaped by both barriers and supports across multiple socioecological levels (Table 2). Participants described individual-level challenges related to limited knowledge, stigma, and competing demands, alongside supports that could facilitate help-seeking and engagement with care. Interpersonal influences included family beliefs and levels of encouragement; organizational factors reflected service structures and resources; community contexts involved social norms and availability of services; and policy environments shaped eligibility and coverage. Taken together, the findings show that caregiver access to mental health support is shaped by conditions operating across multiple parts of the care system rather than at a single level.

#### 3.1.1. Individual-Level Barriers and Supports

Caregivers described barriers that limited awareness of available services, shaped perceptions of whether help-seeking was appropriate, and constrained their ability to access care. These barriers clustered around limited mental health knowledge and literacy, stigma and cultural beliefs, and practical constraints related to caregiving demands. Participants also identified individual-level supports that could reduce these barriers and make help-seeking more feasible.

***Language and literacy barriers.*** Caregivers frequently reported limited awareness of available mental health services and difficulty navigating complex systems. Language barriers were particularly salient among older, Spanish-speaking caregivers, many of whom described challenges using English-dominant mental health systems and digital platforms. Limited English proficiency and lower digital literacy compounded difficulties in identifying services, completing intake processes, and sustaining engagement with care. As one community health worker noted, “They don’t know what websites to search for or what to look for.” Caregivers emphasized that clearer, linguistically appropriate information, step-by-step guidance, and assistance navigating digital platforms could support earlier and more sustained engagement with services.

***Stigma and cultural beliefs.*** Caregivers described internalized beliefs that discouraged acknowledgment of emotional distress and reduced willingness to seek mental health support. Feelings of shame, self-blame, and moral expectations to manage caregiving independently were common. These beliefs contributed to normalization of psychological strain and framing help-seeking as a personal failure rather than a reasonable response to sustained stress. As one caregiver explained, “You should be able to do it all… because that’s what you’re supposed to do.” Others described concealing emotional struggles or seeking support privately due to fear of being perceived as weak or unstable: “You have to go secretly… you don’t have the confidence to say it openly.” Participants noted that education about caregiving-related stress, mental health, and the emotional impacts of sustained caregiving could help normalize distress and support earlier help-seeking.

***Structural Constraints.*** Caregivers described practical barriers that limited access to mental health support, including competing time demands, financial strain, transportation challenges, and immigration-related concerns. High out-of-pocket costs, lost wages, and limited insurance coverage were common obstacles. Undocumented caregivers reported fear that seeking services could jeopardize their legal status. These constraints were especially pronounced among caregivers balancing multiple responsibilities, including those in the “sandwich generation.” Financial barriers were most acute for caregivers with household incomes below USD 25,000, for whom costs related to care, unpaid time off, and transportation posed substantial obstacles to accessing mental health services. Caregivers identified flexible scheduling, low-cost or no-cost services, and support with navigating eligibility, appointments, and logistics as key individual-level supports for overcoming these constraints.

#### 3.1.2. Interpersonal-Level Barriers and Supports

Interpersonal barriers reflected family dynamics and social contexts that shaped caregivers’ willingness and ability to seek mental health support. Family responses influenced whether emotional distress was acknowledged and whether seeking care was viewed as appropriate or necessary. Participants also identified interpersonal supports that could facilitate recognition of caregiver distress and enable engagement with mental health services.

***Family beliefs.*** Many caregivers described family members minimizing or dismissing emotional distress. Mental health concerns were often framed as exaggerated or as something that should be endured privately. As one caregiver noted, “Even in our own Hispanic families… they say, ‘No! You’re fine.’” Caregivers described how these responses reduced validation of their emotional experiences and discouraged open discussion of mental health needs. Participants suggested that family education and open communication about caregiving stress could help legitimize caregivers’ emotional needs and support help-seeking.

***Lack of support or encouragement.*** Caregivers also reported limited emotional or practical support when attempting to seek care. Some described active discouragement, while others emphasized the absence of tangible assistance, such as respite care, that would make attending appointments feasible. One caregiver shared, “I felt very abandoned, very bad.” Caregivers emphasized that practical support from family members, including help with caregiving responsibilities or time to attend appointments, was critical for making mental health care possible.

Taken together, these interpersonal dynamics shaped whether caregivers felt justified in prioritizing their own mental health and whether they were able to initiate and sustain engagement with services. Participants’ accounts highlight the importance of family validation, encouragement, and practical support as key interpersonal conditions for access to mental health care.

#### 3.1.3. Organizational-Level Barriers and Supports

Organizational barriers reflected structural features of health care and social service systems that constrained access even when caregivers recognized a need for mental health support. Participants described how short-term programs, restrictive eligibility criteria, and limited system capacity made it difficult to initiate or sustain care, particularly for caregivers already facing stigma, limited literacy, or family discouragement. At the same time, participants identified organizational practices that could facilitate access when services were stable, flexible, and responsive to caregivers’ needs.

CHWs were frequently described as trusted sources of support for navigating services, highlighting their role as key organizational supports for caregiver mental health access. However, their effectiveness was shaped by organizational constraints. Participants noted variability in CHWs’ mental health training, high caseloads, administrative demands tied to grant-funded programs, and limited authority to address eligibility barriers or service shortages. These conditions restricted sustained follow-up and contributed to emotional strain among CHWs supporting distressed caregivers. Participants emphasized that stronger preparation in caregiver mental health, clearer role definition, and stable funding for CHW positions and caregiver support programs could enhance CHWs capacity to support caregivers with ongoing and evolving needs.

***Short-term and underfunded programs.*** Participants described grant-funded programs as unstable, with services often ending before caregivers could establish consistent support. Continuity of programs was viewed as essential for addressing mental health needs that persist and change over time.

***Restrictive eligibility criteria.*** Caregivers expressed frustration with rigid program requirements that excluded individuals whose needs did not align neatly with eligibility rules. As one caregiver stated, “There needs to be some gray…not everyone fits the qualifications.” Participants noted that greater flexibility in eligibility could better reflect the complexity of caregiving situations and reduce drop-off after initial contact.

***Long wait times.*** Delays in accessing services discouraged follow-through, particularly when caregivers had to arrange alternative care in order to attend appointments. Timely access was described as critical for sustaining engagement once caregivers decided to seek help.

***Lack of cultural responsiveness.*** Participants from racial, ethnic, and sexual minority groups described feeling misunderstood or dismissed by providers. One transgender caregiver shared, “I’m not going to feel comfortable, and my mental health is deteriorating because they are not accepting me as I am.” Culturally and linguistically responsive services were described as necessary for building trust and supporting continued participation in care.

***Employment constraints.*** Caregivers also described workplace policies that limited flexibility for attending mental health appointments. Unsupportive leave policies and concerns about disclosing caregiving responsibilities further constrained access, especially when caregivers felt pressure to share personal information, they preferred to keep private. Flexible scheduling and supportive workplace policies were identified as important supports for making mental health care feasible alongside caregiving responsibilities.

Taken together, these organizational conditions shaped whether caregivers were able to move from recognizing a need for mental health support to successfully initiating and sustaining care.

#### 3.1.4. Community-Level Barriers and Supports

Community-level barriers reflected shared social norms and uneven access to local resources that shaped how caregivers understood mental health and whether support was seen as acceptable or available. Participants described community environments in which stigma, cultural expectations, and limited service availability influenced caregivers’ willingness and ability to seek or sustain mental health care. At the same time, they identified community-level conditions that could support access when mental health needs were visible, normalized, and locally addressed.

***Cultural stigma and community norms.*** Participants described community norms that discouraged open discussion of mental health concerns and framed emotional distress as something to be endured privately. In many settings, professional mental health care was viewed as unnecessary or inappropriate, while prayer, stoicism, or emotional endurance were emphasized instead. Expectations of resilience, particularly for women, further discouraged acknowledgment of distress. As one community health worker explained, “Nobody wants to have a mental health problem…traditionally we tend to hide it.” Participants noted that greater visibility of mental health services and community-based messaging that normalized caregiving stress could help reduce stigma and make help-seeking more acceptable.

***Resource availability.*** Participants also described limited availability of mental health services in certain neighborhoods, particularly in low-income or under-resourced areas. Caregivers perceived that their communities were overlooked in service planning and allocation, which further limited access to care. As one caregiver noted, “Because of the zip code, they think that we don’t need it there—but we do.” Caregivers and CHWs emphasized the importance of accessible, low-cost, and Spanish-language services within local communities, as well as respite options that could support both caregivers and care recipients.

Together, community norms and local resource availability shaped both the perceived acceptability of mental health support and the practical options available to caregivers.

#### 3.1.5. Policy-Level Barriers and Supports

Policy-level barriers reflected structural conditions that shaped caregivers’ eligibility for services and constrained access to mental health support. Participants described how immigration rules, benefit design, and regulatory requirements limited who could receive care and contributed to fear, uncertainty, and delayed help-seeking. At the same time, accounts highlighted how policy conditions could either restrict or enable access depending on how eligibility rules and coverage were defined and implemented.

***Immigration-related restrictions.*** Participants described eligibility rules that excluded undocumented individuals from receiving mental health services, even when need was substantial. One mental health professional noted, “Now anyone who gets our services…has to be legally documented.” These restrictions created fear and uncertainty, discouraging caregivers from seeking care. Caregivers and community health workers serving mixed-status families described how documentation requirements and eligibility checks functioned as barriers within the local service environment, often leading caregivers to avoid or delay contact with mental health systems altogether. Participants noted that policies allowing access regardless of documentation status would reduce fear and make engagement with services more feasible.

***Lack of caregiver-specific coverage.*** Participants emphasized that while mental health services were often available for care recipients, comparable support for caregivers was rarely covered. As one provider explained, “The money is there to provide the service for the individual that’s ill… but the caregiver doesn’t qualify.” This gap in benefit design left caregivers without access to services despite ongoing emotional strain and caregiving demands, reinforcing reliance on informal coping rather than formal support. Participants described caregiver-specific coverage as critical for legitimizing caregivers’ mental health needs and enabling sustained use of services.

Together, policy conditions shaped who was eligible for care, how safe it felt to seek services, and whether mental health support could be accessed consistently over time.

#### 3.1.6. How Barriers Extend Across Levels

Participants described how barriers at one level shaped experiences at others, influencing whether caregivers were able to move from recognizing distress to obtaining care. For example, policy-level eligibility rules limited which services organizations could offer, contributing to program instability and restrictive enrollment criteria. These organizational constraints often resulted in delays or service interruptions, which families interpreted as signals that mental health care was unnecessary or unavailable. Over time, these experiences reinforced caregivers’ tendency to normalize distress and disengage from help-seeking.

Participants also described how limited access to clear, accessible information about mental health services reflected not only individual knowledge gaps but organizational and community-level communication barriers. Difficulties navigating intake systems, eligibility requirements, and digital platforms contributed to uncertainty about where to seek help and whether services were available, reinforcing disengagement across levels. Across stakeholders, delays and failed attempts to access care were described as shaping caregivers’ expectations about the feasibility of mental health support. Repeated experiences of postponement or ineligibility influenced family responses and caregivers’ own perceptions of legitimacy, further reducing follow-through over time.

Together, these accounts illustrate how barriers extended across levels through everyday service encounters rather than occurring as isolated obstacles. While participants described supports such as navigation assistance and trusted community-based relationships, these supports were often embedded within the same organizational and policy constraints that limited service availability, reducing their capacity to interrupt the cross-level processes described above.

## 4. Discussion

Guided by the Socioecological Model, this qualitative study examined barriers to and supports for mental health access among informal caregivers in Harris County, Texas, drawing on perspectives from caregivers, community health workers, and mental health professionals. Rather than locating access challenges solely at the individual level, the findings demonstrate how both barriers and supports are shaped through reinforcing interactions among service infrastructure, organizational practices, community norms, and policy environments. Experiences of stigma, fragmented services, and eligibility constraints were embedded within institutional and community contexts that influenced caregivers’ ability to recognize mental health needs, navigate available services, and sustain engagement with care, while identified supports such as community health worker navigation and culturally responsive services were often limited by structural instability. By situating caregiver experiences within organizational, community, and policy contexts and integrating multiple stakeholder perspectives, this study extends prior research that has focused primarily on individual or interpersonal barriers and identifies system-level leverage points for intervention across service delivery, community navigation, and policy design.

At the individual level, caregivers identified stigma, emotional self-reliance, and limited mental health literacy as barriers to recognizing distress and seeking support. Culturally grounded beliefs and moral expectations surrounding caregiving often normalized emotional strain and framed help-seeking as unnecessary or inappropriate, even amid significant burden. These findings are consistent with prior research documenting internalized stigma and reluctance to seek mental health care among caregivers [18,22,23,24]. Participants also described practical constraints, including time demands, financial strain, and language and technology barriers, which limited their ability to act on perceived need, even when distress was recognized.

At the interpersonal level, family responses often minimized caregivers’ emotional distress or discouraged care-seeking. Consistent with prior work on relational invisibility in caregiving [17,25], caregivers reported feeling dismissed when expressing mental health concerns. Family dynamics shaped whether distress was viewed as legitimate and whether seeking care was supported, contributing to delays in engagement with mental health services. Beyond family contexts, organizational conditions further constrained access. Caregivers and providers described long wait times, restrictive eligibility criteria, and limited cultural responsiveness, reflecting broader behavioral health access inequities [26]. Community health workers emphasized the instability of short-term, grant-funded programs as a recurring barrier to continuity and trust. Program instability constrained sustained engagement, particularly for caregivers with ongoing and evolving needs, highlighting that access depends not only on service availability but also on durability and workforce stability.

At the community level, social norms reinforced expectations of emotional endurance and stigmatized mental health concerns, particularly for women. Participants described environments in which vulnerability was viewed as weakness and professional mental health care as unnecessary or inappropriate. These findings underscore the role of community narratives in shaping the perceived acceptability of mental health support and align with prior public health research on stigma and social norms [27]. At the policy level, immigration-related eligibility restrictions and the absence of caregiver-specific coverage excluded many caregivers from mental health services despite substantial need. These barriers reflected how federal eligibility rules were locally interpreted and implemented within Harris County. While policy environments vary across jurisdictions, the findings illustrate how local policy implementation can function as a structural constraint on caregiver mental health access in restrictive settings.

Taken together, these findings demonstrate that barriers to caregiver mental health support operate across socioecological levels and accumulate over time. Service delays, eligibility exclusions, and limited program stability discouraged follow-through and contributed to normalization of distress and disengagement from care. From a public health perspective, the findings highlight the importance of addressing service delivery, community norms, and policy environments alongside individual-level supports for caregiver mental health. Although grounded in a single urban setting, the findings offer insight into processes that may similarly constrain access to caregiver mental health support in other under-resourced contexts.

### 4.1. Implications for Practice and Policy

The results of this study underscore the need for approaches to caregiver mental health that extend beyond individual help-seeking and address service design, community support, and policy environments. Participants’ accounts indicate that efforts to reduce stigma and increase engagement with mental health services are more likely to be effective when they are culturally and linguistically appropriate and responsive to caregivers’ limited time, high caregiving demands, and competing responsibilities. Practical strategies aligned with these needs include caregiver-focused mental health screening, bilingual navigation support, and delivery of mental health information in settings caregivers already access, such as community organizations and health care sites.

Accounts across stakeholder roles also highlight the limitations of short-term and fragmented service models for caregivers whose mental health needs are ongoing and often intensify over time. CHWs emerged as critical supports for navigating eligibility rules, scheduling appointments, and identifying available services, particularly for caregivers facing language barriers, financial strain, or immigration-related concerns. At the same time, their capacity to provide sustained assistance was constrained by limited mental health training and unstable funding. These observations point to the importance of organizational investments that support CHW training in mental health navigation, integration within care teams, and funding structures that promote continuity rather than episodic support.

Policy contexts further shaped caregivers’ access to mental health services. Gaps in benefit design frequently excluded caregivers even when care recipients qualified for services, and immigration-related restrictions contributed to fear and delayed help-seeking. Policy approaches that explicitly recognize caregivers as having mental health needs of their own and that support community-based service delivery may reduce these barriers. Taken together, the implications suggest that improving caregiver mental health requires changes to how services are organized, delivered, and funded, rather than relying primarily on efforts to modify individual attitudes or behaviors.

### 4.2. Strengths and Limitations

This study has several strengths. Use of the Socioecological Model supported a system-focused examination of caregiver mental health access across individual, interpersonal, organizational, community, and policy contexts. Inclusion of informal caregivers, CHWs, and mental health professionals provided complementary perspectives from different points within caregiving and service systems and supported analytic triangulation. Methodological rigor was strengthened through independent coding, team-based consensus procedures, and use of analytic memos and audit trails. The inclusion of participants from diverse cultural, linguistic, and socioeconomic backgrounds also strengthened the depth and interpretive scope of the findings.

Several limitations should also be noted. First, purposive and snowball sampling may have influenced the range of perspectives represented and may have favored caregivers who were more connected to services or social networks, potentially underrepresenting more isolated caregivers. Future studies could broaden recruitment through primary care settings, community health workers, social media, and local organizations to reach caregivers with varying levels of connectivity.

Second, the sample size was appropriate for in-depth qualitative analysis but limits transferability to broader caregiver populations. More than half of caregivers were supporting individuals with cancer, which may have emphasized cancer-related stressors, service pathways, and interactions with health care institutions. Caregivers supporting individuals with other conditions, such as dementia, cardiovascular disease, or neurological disorders, may face different caregiving demands and service gaps.

Third, the demographic composition of the sample, which included a predominance of Hispanic, low-income, and bilingual participants, likely increased the prominence of language access, digital exclusion, and immigration-related barriers. While these characteristics reflect the study setting and are common in many under-resourced urban areas, the findings may be less applicable to caregivers with higher socioeconomic status, greater English proficiency, or stable access to employer-based mental health benefits.

Fourth, interpretation of policy-related barriers should be understood within the regulatory, funding, and implementation context of Texas. Caution is warranted in extending these findings to jurisdictions with more expansive caregiver support or immigrant health policies. Finally, the study was conducted in a single large urban area with an established network of community-based organizations. Findings may not translate to rural or remote settings, where caregivers often face additional challenges such as geographic isolation, limited availability of services, and fewer community-based resources.

## 5. Conclusions

Caregivers play a vital role in sustaining health care delivery, yet this study highlights the substantial and often under-addressed barriers they face in accessing mental health support. This study contributes qualitative evidence that caregivers’ access to mental health support is shaped by service design, eligibility rules, and local care systems, not solely by individual awareness or motivation. By examining caregiver experiences alongside those of CHWs and mental health professionals, the findings show how features of mental health service organization influence whether caregivers are able to obtain support when needed. Taken together, the results underscore the importance of treating caregiver mental health access as a health systems and public health issue. Improving caregiver well-being will require attention to how mental health services are organized, funded, and delivered, as well as to the supports available to help caregivers navigate those systems. Future research should examine caregiver mental health across diverse care settings and policy contexts to inform approaches that better align services with caregivers’ needs.

## Figures and Tables

**Table 1:** Participant Characteristics.

Characteristic	All *n* = 51	Informal Caregivers *n* = 17	CHWs *n* = 23	Mental Health Professionals *n* = 11
Age (mean in years)	45.9	48.6	45.0	43.7
Gender				
Man	8 (15.7)	4 (23.5)	2 (8.7)	2 (18.2)
Female	40 (78.4)	13 (76.5)	18 (78.3)	9 (81.8)
Transgender woman	3 (5.9)	0	3(13.0)	0
Sexual Orientation				
Straight	38 (74.5)	13 (76.5)	15 (65.2)	10 (90.9)
Gay	10 (19.6)	3 (17.6)	6 (26.1)	1 (9.1)
Bisexual	2 (3.9)	0	2 (8.7)	0
Not reported	1(2.0)	1 (5.9)	0	0
Relationship Status				
Single	12 (23.6)	4 (23.5)	6 (26.1)	2 (18.2)
Married/Cohabitating	30 (58.8)	11 (64.7)	10 (43.5)	9 (81.8)
Divorced/Separated	4 (7.8)	1 (5.9)	3 (13.0)	0
Widowed	1 (2.0)	1 (5.9)	0	0
Prefer not to answer	4 (7.8)	0	4 (17.4)	0
Hispanic				
Yes	38 (74.5)	14 (82.4)	20 (87.0)	4 (36.4)
No	13 (25.5)	3 (17.6)	3 (13.0)	7 (63.6)
Race				
White	40 (78.4)	14 (82.4)	20 (87.0)	6 (54.5)
Black/African American	9 (17.6)	3 (17.6)	2 (8.7)	4 (36.4)
Asian American	1 (2.0)	0	0	1 (9.1)
Prefer not to answer	1 (2.0)	0	1 (4.3)	0
Education				
Less than high school	2 (3.9)	2 (11.8)	0	0
Some high school	3 (5.9)	0	3 (13.0)	0
High school diploma/GED	18 (35.3)	9 (52.9)	9 (39.2)	0
Associate’s/Technical	5 (9.8)	2 (11.8)	3 (13.0)	0
Bachelor’s degree	8 (15.7)	3 (17.6)	4 (17.4)	1 (9.1)
Graduate degree	13 (25.5)	0	3 (13.0)	10 (90.9)
Prefer not to answer	2 (3.9)	1 (5.9)	1 (4.4)	0
Household Income				
Less than $15,000	6 (11.8)	2 (11.8)	4 (17.4)	0
$15,000–$24,999	9 (17.6)	5 (29.4)	4 (17.4)	0
$25,000–$49,999	11 (21.6)	1 (5.9)	9 (39.1)	1 (9.1)
$50,000 and above	15 (29.4)	3 (17.6)	2 (8.7)	10 (90.9)
Prefer not to answer	10 (19.6)	6 (35.3)	4 (17.4)	0

**Table 2:** Multi-level Barriers in Seeking and Obtaining Mental Health Support: Themes by Level of the Socioecological Model.

SEM Level	Themes
Individual	Knowledge and literacy (low mental health literacy, language barriers, digital exclusion); Stigma and cultural beliefs (shame, pride, caregiving duty, stigma); Structural constraints (cost, time demands, transportation challenges, immigration-related fears)
Interpersonal	Family beliefs (mental health minimized, prayer/stoicism promoted); Lack of support or encouragement (emotional discouragement, absence of practical support such as respite care)
Organizational	Short-term/underfunded programs; Restrictive eligibility criteria; Long wait times; Lack of cultural competence; Employment constraints (unsupportive leave policies, disclosure pressures)
Community	Cultural stigma (silence, resilience expectations); Resource availability (limited services in underserved areas, geographic disparities)
Policy	Immigration policies (eligibility restrictions for undocumented individuals); Lack of caregiver-specific support or coverage

## Data Availability

De-identified data from this study are not publicly available due to institutional IRB restrictions. Data may be made available upon reasonable request to the corresponding author, subject to IRB approval.

## Abbreviations

The following abbreviations are used in this manuscript:

CHW	Community Health Worker
SEM	Socioecological Model
